# A comparative cross-platform analysis of cuproptosis-related genes in human nonobstructive azoospermia: An observational study

**DOI:** 10.1097/MD.0000000000039176

**Published:** 2024-08-02

**Authors:** Silin Jiang, Yong Wei, Yongshan Li, Wei Liu, Zhenzhong Wang, Xuhui Meng, Qingyi Zhu, Luming Shen

**Affiliations:** a Department of Urology, The Second Affiliated Hospital of Nanjing Medical University, Nanjing, China.

**Keywords:** bioinformatic analysis, cuproptosis, differential expressed genes, nonobstructive azoospermia

## Abstract

This study aimed to identify novel biomarkers associated with cuproptosis in human nonobstructive azoospermia (NOA). We obtained 4 NOA microarray datasets (GSE145467, GSE9210, GSE108886, and GSE45885) from the NCBI Gene Expression Omnibus database and merged them into training set. Another NOA dataset (GSE45887) was used as validation set. Differentially expressed cuproptosis-related genes were identified from training set. Gene Ontology function and Kyoto Encyclopedia of Genes and Genomes pathway analyses were conducted. Least absolute shrinkage and selection operator regression and support vector machine-recursive feature elimination were used to identify hub cuproptosis-related genes. We calculated the expression of the hub cuproptosis-related genes in both validation set and patients with NOA. Gene set variation analysis was used to explore their potential biological functions. The risk prediction model was built by logistic regression analysis and was evaluated in the validation set. Finally, we constructed a competing endogenous RNA network. The training set included 29 patents in the control group and 92 in the NOA group, and 10 cuproptosis-related differentially expressed genes were identified. Subsequently, we screened 6 hub cuproptosis-related genes (DBT, GCSH, NFE2L2, NLRP3, PDHA1, and SLC31A1) by least absolute shrinkage and selection operator regression and support vector machine-recursive feature elimination. GCSH, NFE2L2, NLRP3, and SLC31A1 expressed higher in NOA group than in control group (*P* < .05) in the validation set (4 patients in control and 16 in NOA groups), while the expression levels of GCSH, NFE2L2, NLRP3, PDHA1, and SLC31A1 were higher in NOA group than in control group (*P* < .05) in our patients (3 patients in control and 4 in NOA groups). The model based on the 6-gene signature showed superior performance with an AUC value of 0.970 in training set, while 1.0 in validation set. Gene set variation analysis revealed a higher enrichment score of “homologous recombination” in the high expression groups of the 6 hub genes. Finally, we constructed a competing endogenous RNA network and found hsa-miR-335-3p and hsa-miR-1-3p were the most frequently related to the 6 hub genes. DBT, GCSH, NFE2L2, NLRP3, PDHA1, and SLC31A1 may serve as predictors of cuproptosis and play important roles in the NOA pathogenesis.

## 1. Introduction

Nonobstructive azoospermia (NOA) is a severe form of male infertility characterized by the absence of sperm in the semen.^[1]^ Unlike obstructive azoospermia, which occurs when sperm flow is blocked, NOA is primarily caused by problems in the production or maturation of sperm cells within the testes. NOA is estimated to accounts for 10–15% of male infertility cases, affecting approximately 1% of all men.^[2]^ Several potential factors can contribute to the development of nonobstructive azoospermia, including genetic abnormalities, hormonal imbalances, testicular injury or damage, certain medications, and medical conditions such as Klinefelter syndrome.^[3]^ Gonadotropin therapy is clinically effective for azoospermia due to hypogonadotropin hypogonadism, but drug therapy is generally ineffective for azoospermia due to other causes.^[4]^

Continuous advancements in second-generation sequencing technology have significantly contributed to our understanding of the genetic basis and progression of various diseases, leading to the identification of an increasing number of potential biomarkers and molecular mechanisms.^[5]^ In the context of spermatogenesis and male infertility, researchers have extensively studied the expression patterns of mRNAs, miRNAs, and lncRNAs in testicular specimens obtained from NOA patients. The primary objective was to identify differentially expressed genes and explore their molecular functions.^[6–9]^ Investigating the expression profiles of these RNA molecules in testicular tissue holds great promise for uncovering the underlying molecular mechanisms involved of NOA.

Copper plays a central role in many important biological processes, such as mitochondrial respiration, antioxidant defense, and biocompound synthesis.^[10]^ Recently, cuproptosis, a newly identified mechanism of cellular death, has been discovered. Cuproptosis occurs when there is an abnormal accumulation of free copper and protein lipidation within cells. This aberrant process leads to cytotoxic stress, which ultimately results in cell death.^[11]^ Previous studies have demonstrated and confirmed the toxic effects of copper on spermatozoa.^[12,13]^ However, the occurrence of cuproptosis in the context of NOA has not yet been reported.

The machine learning method aims to conduct a preliminary discussion on the potential molecular signal pathway by utilizing bioinformatics technology to comprehensively assess multiple databases for modeling purposes, thereby assisting in the diagnosis and prognosis of diseases, including NOA. Previous studies have employed various machine learning methods, such as the Boruta algorithm, least absolute shrinkage and selection operator (LASSO) regression, and support vector machine-recursive feature elimination (SVM-RFE), to identify diagnostic signature genes in NOA.^[14–16]^

This study aimed to investigate the potential involvement of cuproptosis in NOA. We analyzed 4 RNA expression profiling microarrays obtained from the NCBI Gene Expression Omnibus (GEO) database, specifically focusing on individuals with NOA and control individuals with normal spermatogenesis. We integrated the microarray data with the cuproptosis-related genes (CRGs) previously reported by Tsvetkov et al.^[11]^ We conducted differential gene analysis, followed by Gene Ontology (GO) function analysis and Kyoto Encyclopedia of Genes and Genomes (KEGG) pathway analysis on these differentially expressed genes. Additionally, we employed machine learning algorithms to identify hub CRGs. Furthermore, we performed a gene set variation analysis (GSVA) to explore their potential biological functions. The construction of the ceRNA network and the diagnostic model in this study provides a solid foundation for further research in this field.

## 2. Methods

### 2.1. Data source and normalization

**Figure 1.**
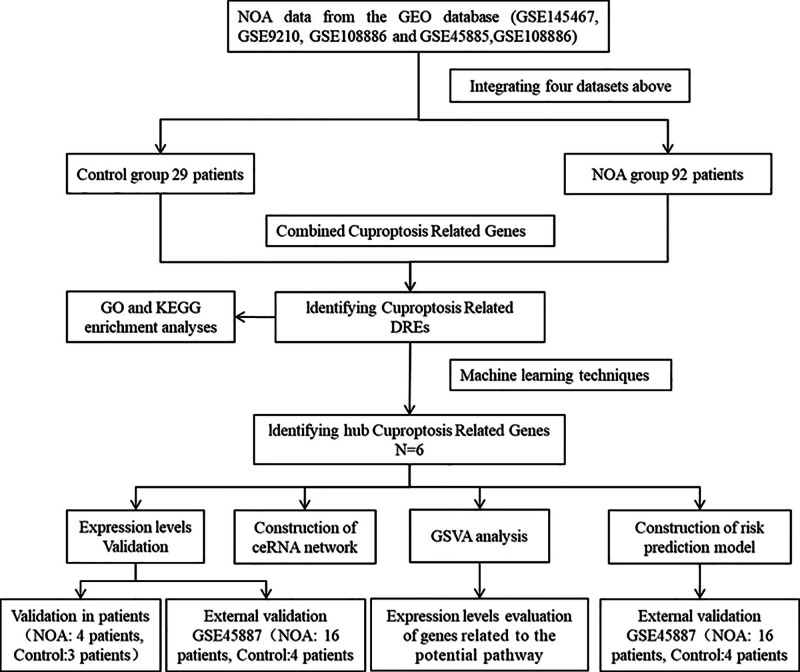
The diagrammatic illustration showing the workflow for the identification of hub CRGs in NOA. CRGs = cuproptosis-related genes, NOA = nonobstructive azoospermia.

In our study, we downloaded 5 microarray datasets related to NOA from the GEO database (https://www.ncbi.nlm.nih.gov/geo/). A diagrammatic illustration of our research approach is shown in Figure 1. The specific datasets utilized were GSE145467, GSE9210, GSE108886, GSE45885, and GSE45887. The characteristics of the datasets are listed in Table 1. To ensure the comparability and eliminate any batch effects, we used the “sva” package to remove differences between batches. We combined the gene expression matrices from GSE145467, GSE9210, GSE108886, and GSE45885 to create a new matrix, which we referred to as the training set. In the final combined dataset, there were 29 samples in the control group and 92 in the NOA group. Additionally, we used GSE45887 as the validation set.

**Table 1:** List of microarray datasets included in the study obtained from GEO database for nonobstructive azoospermia.

GEO Accession	Subject			Sample	Platform
	Control	NOA	Total		
GSE145467	10	10	20	Testis tissue	GPL4133
GSE9210	11	47	58	Testis tissue	GPL887
GSE45885	4	27	31	Testis tissue	GPL6244
GSE108886	4	8	12	Testis tissue	GPL10558
GSE45887	4	16	2	Testis tissue	GPL6244

### 2.2. Identification of differentially expressed CRGs and functional enrichment analysis

In our study, we obtained a total of 19 CRGs from a previous study.^[11]^ We then combined the expression data to create an expression matrix specifically for CRGs. Next, we utilized the “limma” package to identify differentially expressed CRGs. These genes were identified based on 2 criteria: an adjusted *P* value < .05 and an absolute log2 fold change (FC) > 0.3. Using these differentially expressed CRGs, we conducted comprehensive enrichment analyses utilizing GO, and KEGG using the R package “clusterProfiler.”

### 2.3. Identification of cuproptosis-related hub genes by machine learning methods

To identify associated genes related to cuproptosis, we employed 2 machine learning approaches: LASSO regression analysis using the “glmnet” package in R, and support vector machine-recursive feature elimination (SVM-RFE) machine learning technique. The SVM module is built by the “e1071” package. Both of these machine learning approaches helped us identify the hub CRGs for further analysis.

### 2.4. Evaluation of hub CRGs expression levels

We evaluated the expression levels of hub CRGs in the validation set and our NOA patients. Testicular biopsy samples were collected from 4 patients with NOA who underwent diagnostic testicular biopsy after ruling out various known causes of infertility, such as anti-antibodies, Y chromosome microdeletions, varicocele, chromosomal aberrations, orchitis, and testicular torsion. Additionally, normal samples were obtained from 3 men with normal spermatogenesis who underwent orchiectomy for testicular tumors. Total RNA was extracted from the testis samples using TRIzol reagent (Invitrogen, Carlsbad, CA) and quantified using a spectrophotometer (Applied Biosystems, Foster City, CA). Quantitative reverse transcription polymerase chain reaction (qRT-PCR) was performed on a StepOne Real-Time PCR machine (Applied Biosystems) using iTaqTM SYBR Green Supermix with ROX (Bio-Rad, Hercules, CA). The mRNA levels were normalized to the housekeeping gene GAPDH using the ∆∆Ct method. Primer sequences are listed in Supplementary Table S1, Supplemental Digital Content, http://links.lww.com/MD/N298.

### 2.5. Nomogram construction and assessment of diagnostic efficacy

We constructed a risk prediction model based on the identified hub CRGs and visualized it using a nomogram. Subsequently, a ROC curve was plotted and the AUC value was computed using the “pROC” R package. Additionally, the AUC and calibration curve were utilized for a comprehensive evaluation of the diagnostic efficacy. Finally, the predictive reliability of the hub CRGs was further validated using the validation set.

### 2.6. Gene set variation analysis (GSVA) of hub CRGs

We utilized the “GSVA” R package to identify the pathways most associated with the hub CRGs. The reference gene sets used in the GSVA were obtained from the Molecular Signatures Database, specifically the “c2.cp.kegg.v6.2.symbols.gmt” dataset. This analysis allowed us to gain insights into the biological functions and pathways associated with the hub genes.

### 2.7. LncRNA-miRNA prediction of hub CRGs

We used databases, including miRanda, miRDB, and TargetScan, to identify potential miRNAs that may target hub genes. Similarly, the SpongeScan database was used to identify lncRNAs that could potentially interact with the hub genes. Subsequently, we constructed a competing endogenous RNA (ceRNA) network using the identified miRNAs and lncRNAs. This network provides a comprehensive understanding of the potential regulatory interactions among the hub genes, miRNAs, and lncRNAs.

### 2.8. Ethics approval

Studies were reviewed and approved by the Ethics Committee of the Second Affiliated Hospital of Nanjing Medical University (the Ethical Approval No. 2023-KY-050-01).

### 2.9. Statistical analysis

Statistical analysis was performed using R software v4.1.0. Spearman analysis was used to calculate the correlation coefficients. Two-tailed Student *t* test or Mann–Whitney *U* test was used to compare the expression of differential genes between the NOA and control groups. Logistic regression analysis was used to constructed the risk prediction model. All results with a *P* value < .05 were considered statistically significant.

## 3. Results

### 3.1. Identification of differentially expressed CRGs and functional enrichment analysis

**Figure 2.**
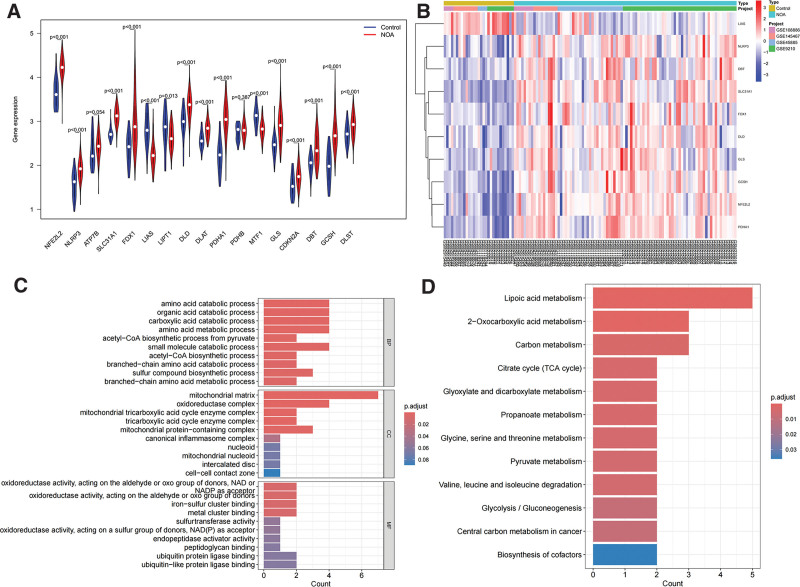
Identification of differentially expressed CRGs between the NOA group and the control group. (A) The expression levels of CRGS. Nine genes (DBT, DLD, FDX1, GCSH, GLS, NFE2L2, NLRP3, PDHA1, and SLC31A1) were significantly higher in NOA group than in control group (*P* < .05), whereas LIAS was higher in control group (*P* < .001). (B) Heatmap. (C) GO enrichment analysis. (D) KEGG enrichment analysis of differentially expressed CRGs. CRGs = cuproptosis-related genes, GO = Gene Ontology, KEGG = Kyoto Encyclopedia of Genes and Genomes, NOA = nonobstructive azoospermia.

The GSE145467, GSE9210, GSE108886, and GSE45885 datasets were merged to obtain the batch-corrected data. We then merged the expression matrix with the CRGs to obtain an expression matrix specifically for CRGs. The volcano map and heatmap showed 10 (52.63%) differentially expressed CRGs between the NOA and control groups (Fig. 2A and B, *P* < .05). In the NOA group, 9 genes (DBT, DLD, FDX1, GCSH, GLS, NFE2L2, NLRP3, PDHA1, and SLC31A1) were significantly higher in NOA group than in control group (*P* < .05), whereas LIAS was higher in control group (*P* < .001). GO enrichment analysis revealed that the differentially expressed genes were enriched in terms such as “oxidoreductase activity,” “mitochondrial tricarboxylic acid cycle enzyme complex,” “iron–sulfur cluster binding,” and “metal cluster binding” (Fig. 2C). KEGG enrichment analysis showed that these genes were associated with pathways such as the “Citrate cycle (TCA cycle)” and “Lipoic acid metabolism” (Fig. 2D).

### 3.2. Identification of cuproptosis-related hub genes by machine learning methods

**Figure 3.**
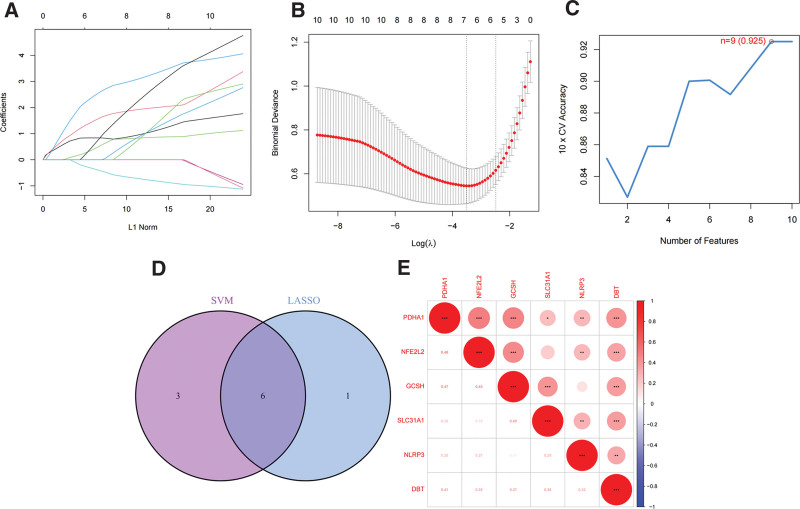
Screening of hub CRGs. (A, B) LASSO regression for screening hub genes. (C) SVM-RFE algorithm for screening hub genes. (D) Venn diagram showing the intersection of the genes obtained by both algorithms. (E) Correlation analysis of the hub CRGs. CRGs = cuproptosis-related genes.

In our study, we employed 2 machine learning algorithms, LASSO regression and SVM-RFE, to screen for hub genes from the ten differentially expressed CRGs, respectively. LASSO regression identified 7 (36.84%) CRGs (Fig. 3A and B), whereas the SVM-RFE algorithm identified 9 (47.37%) CRGs (Fig. 3C). Notably, 6 (31.58%) genes (DBT, GCSH, NFE2L2, NLRP3, PDHA1, and SLC31A1) were consistently identified using both algorithms (Fig. 3D). The intergenic correlations between the 6 genes was also analyzed (Fig. 3E).

### 3.3. Expression levels of hub CRGs

**Figure 4.**
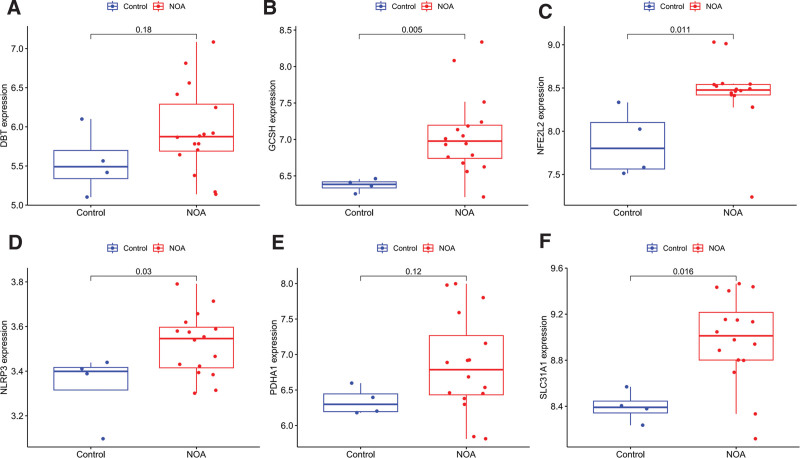
The expression of 6 hub CRGs was evaluate in the validation set. (A) DBT, (B) GCSH, (C) NFE2L2, (D) NLRP3, (E) PDHA1, and (F) SLC31A1.The expression levels of GCSH, NFE2L2, NLRP3, and SLC31A1 were significantly higher in NOA patients than in control patients (*P* < .05). CRGs = cuproptosis-related genes, NOA = nonobstructive azoospermia.

**Figure 5.**
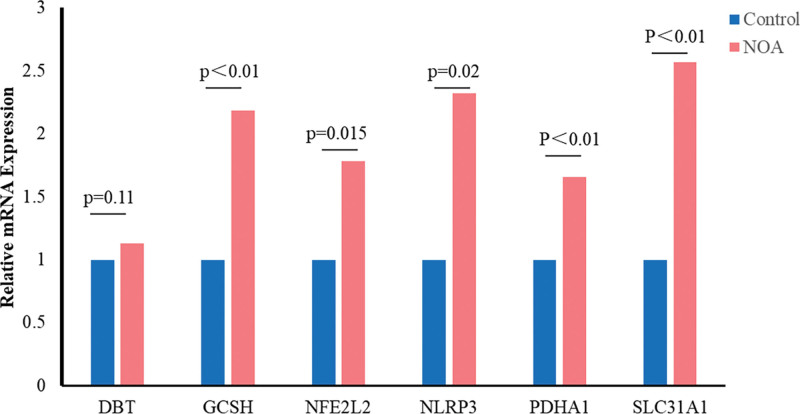
The expression of 6 hub CRGs was evaluated in our NOA and control patients. The expression levels of GCSH, NFE2L2, NLRP3, PDHA1, and SLC31A1 were statistically higher than in control patients (*P* < .05). CRGs = cuproptosis-related genes, NOA = nonobstructive azoospermia.

The expression of the 6 hub genes was also evaluated in the validation set as well (Fig. 4A–F). The expression levels of GCSH, NFE2L2, NLRP3, and SLC31A1 were significantly higher in NOA patients than in control patients (*P* < .05). Although the expression levels of PDHA1 and DBT did not statistically indicated relatively higher expression in patients with NOA. In our NOA patients (Fig. 5), the expression levels of GCSH, NFE2L2, NLRP3, PDHA1, and SLC31A1 were statistically higher than in control patients (*P* < .05), but there was no significant difference in the expression of DBT between the 2 groups (*P* = .11).

### 3.4. Nomogram construction and assessment of diagnostic efficacy

**Figure 6.**
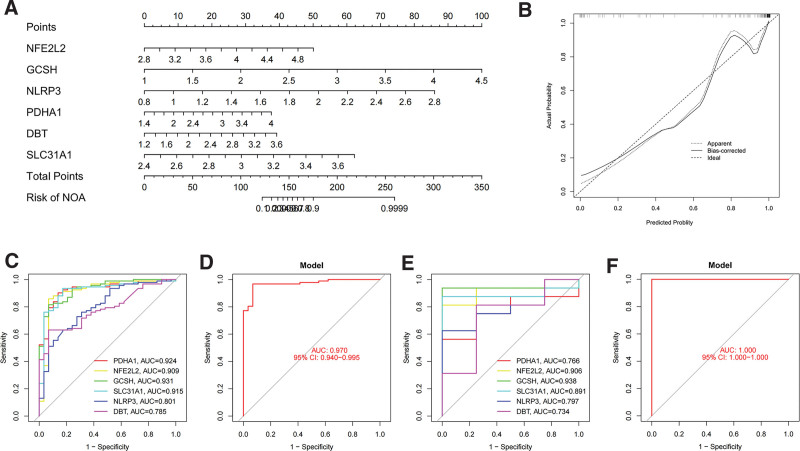
The prediction model of the 6 hub CRGs. (A) Construction of a nomogram to predict NOA risk based on the 6 hub CRGs model. (B) Calibration curves to assess the predictive ability of the nomogram model. (C, D) ROC of the 6 hub CRGs model in training set. (E, F) ROC of the 6 hub CRGs model in validation set. CRGs = cuproptosis-related genes, NOA = nonobstructive azoospermia.

This model was visualized as a nomogram (Fig. 6A and B). Receiver operating characteristic (ROC) analysis was also performed to compare the predictive accuracy of the model. We observed that our model was optimal, with the highest AUC value of up to 0.970, compared to other single biomarker models (Fig. 6C and D). The sensitivity, accuracy, and specificity of ROC analysis were 0.967, 0.959, and 0.931, respectively. These results were verified using the validation set (Fig. 6E and F).

### 3.5. GSVA of hub CRGs

**Figure 7.**
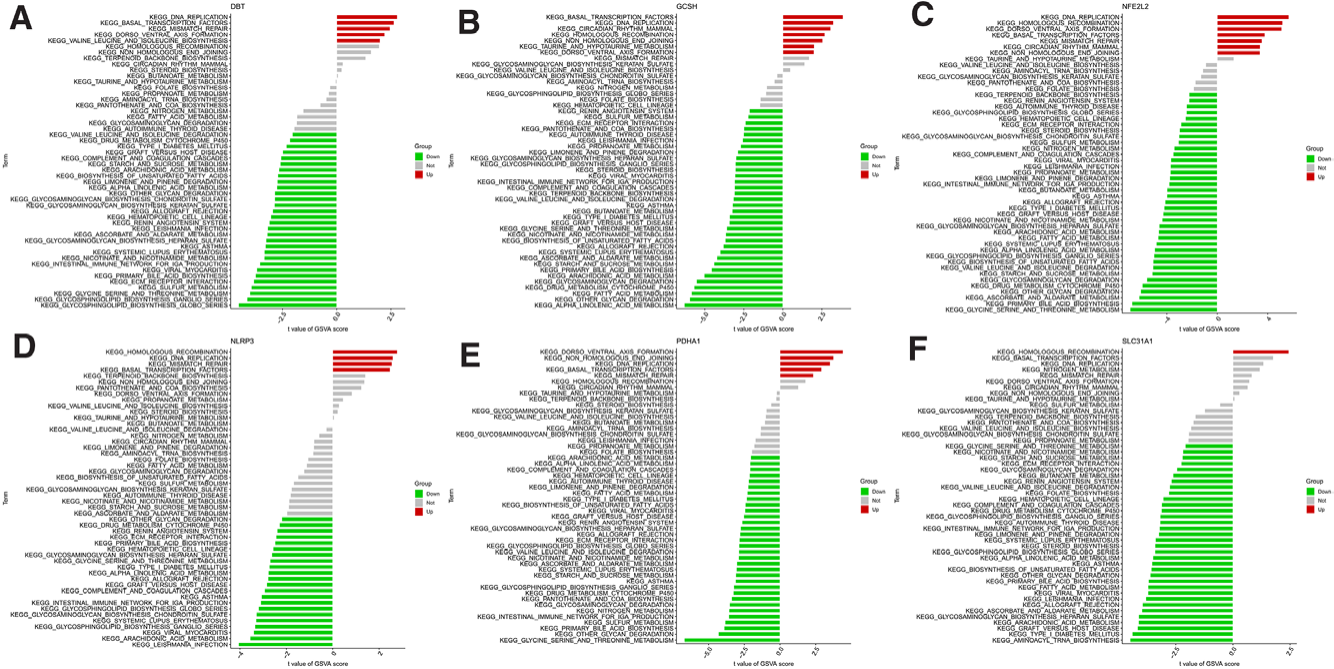
GSVA analysis of the hub CRGs. Differentially expressed pathways of single hub CRG were displayed. (A) DBT, (B) GCSH, (C) NFE2L2, (D) NLRP3, (E) PDHA1, and (F) SLC31A1. CRG = cuproptosis-related gene, NOA = nonobstructive azoospermia.

**Figure 8.**
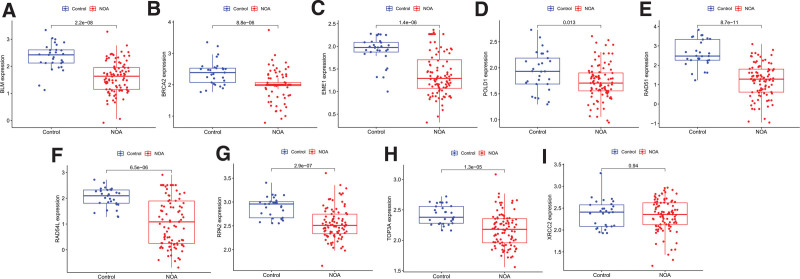
The expression of the genes related to “homologous recombination” pathway was evaluate in the training set. (A) BLM, (B) BRCA2, (C) EME1, (D) POLD1, (E) RAD51, (F) RAD54L, (G) RPA2, (H) TOP3A, and (I) XRCC2. The expression levels of BLM, BRCA2, EME1, POLD1, RAD51, RAD54L, RPA2, and TOP3A were lower in the NOA group than in the control group (*P* < .05). NOA = nonobstructive azoospermia.

The results of GSVA showed that “homologous recombination” exhibited a higher enrichment score in the high expression groups of hub CRGs (Fig. 7A–F). We analyzed the expression levels of genes related to “homologous recombination” in microarray datasets related to NOA (Fig. 8A–J). Most of these genes (88.8%), including BLM, BRCA2, EME1, POLD1, RAD51, RAD54L, RPA2, and TOP3A, expressed lower in the NOA group than in the control group (*P* < .05).

### 3.6. ceRNA prediction of hub CRGs

**Figure 9.**
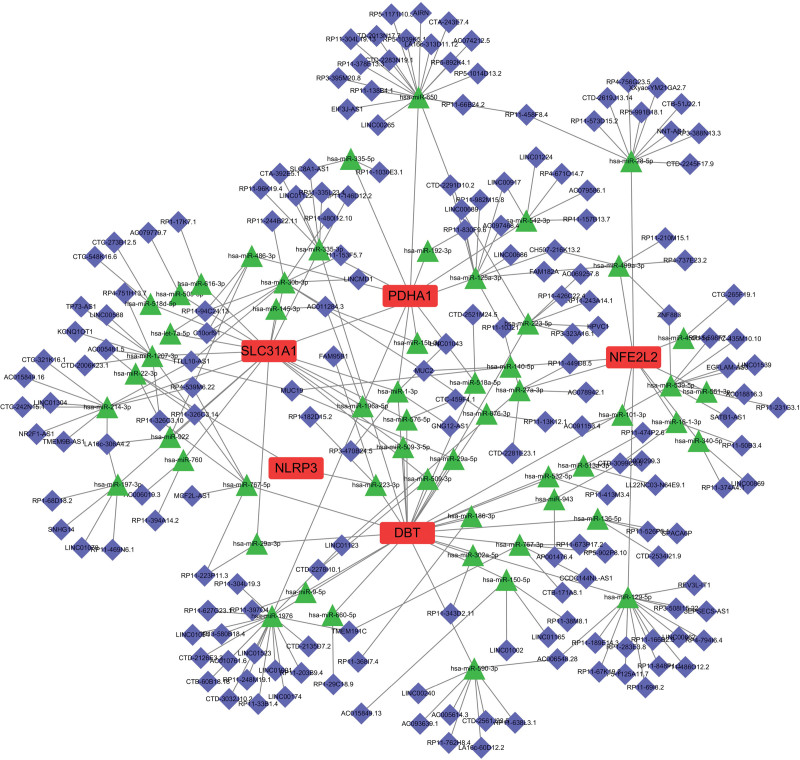
The ceRNA network of the 6 hub CRGs. ceRNA = competing endogenous RNA, CRGs = cuproptosis-related genes.

To gain insight into the regulatory mechanisms of noncoding RNAs on hub genes, we conducted an extensive search across various online databases. Our search yielded 55 miRNAs and 177 lncRNAs that potentially regulate the hub genes (Fig. 9). Among these regulatory molecules, hsa-miR-335-3p and hsa-miR-1-3p were found to be associated with 3 hub genes. Specifically, hsa-miR-1-3p is regulated by the noncoding RNAs LINC01043, RP3-470B24.5, and GNG12-AS1. Hsa-miR-335-3p is regulated by SLC8A1-AS1, CTA-392E5.1, RP11-146D12.2, RP11-335L23.4, LINC01122, and RP11-96K19.4.

## 4. Discussion

In the current study, gene expression profiles of NOA samples from 4 GEO database were assessed. We obtained 29 samples in the control group and 92 in the NOA group. Initially, ten cuproptosis-related differentially expressed genes were identified. Additionally, these genes were enriched in various pathways, such as oxidoreductase activity and mitochondrial tricarboxylic acid cycle enzyme complex. Subsequently, 6 hub CRGs (DBT, GCSH, NFE2L2, NLRP3, PDHA1, and SLC31A1) were identified by Machine learning methods, each with an AUC value exceeding 0.78.

The deleterious effect of copper on spermatozoa was first demonstrated by Quatrefages in 1950.^[17]^ Cuproptosis, a recently identified form of non-apoptotic cell death, stands apart from established modes like apoptosis, necrosis, pyroptosis, and ferroptosis. This process arises from the disruption of mitochondrial respiration, primarily triggered by the excessive binding of copper to the lipid-acylated components of the tricarboxylic acid cycle. Consequently, lipid-acylated-related proteins aggregates and iron-sulfur cluster proteins are lost, and intracellular proteotoxic stress ensues, ultimately leading to cell death.^[11]^ More than half of CRGs were differentially expressed between NOA group and control group, indicating that CRGs play a significant role in the development of NOA.

The DBT gene encodes dihydrolipoamide branched-chain transacylase E2, which plays a crucial role as a vital component of the branched-chain alpha-keto acid dehydrogenase complex (BCKDH).^[18]^ Its main function involves the transfer of acyl groups from the BCKDH complex to coenzyme A (CoA). CoA is an indispensable co-factor that participates in numerous metabolic pathways, including the TCA cycle. Protein lipoylation is an extensively conserved posttranslational modification that primarily targets specific lysine residues. This modification has been observed in only 4 enzymes, namely DBT, GCSH, DLST, and DLAT.^[19]^ Notably, the knockout of any of these lipoylation-related enzymes has been shown to effectively protect cells from copper toxicity.^[11]^ Thus, the high expression of DBT in testicular tissue may be more likely to be influenced by cuproptosis.

GCSH is the gene responsible for encoding the enzyme glycine cleavage system H protein. This enzyme serves as a crucial component of the glycine cleavage system and plays a vital role in glycine metabolism.^[20]^ The glycine cleavage system is involved in glutathione synthesis. Recent evidence has shed light on the significant role of glutathione in the regulation of copper entry and intracellular copper homeostasis.^[21]^ Previous studies have found that decreased glutathione levels lead to oligospermia and azoospermia.^[22,23]^ Thus, GCSH expression may be upregulated to mitigate cell damage when cuproptosis occurs.

NFE2L2, also referred to as Nrf2 (nuclear factor erythroid 2-related factor 2), encodes a vital transcription factor involved in cellular defense against oxidative stress. Nrf2 knockout results in increased spontaneous apoptosis and heightened susceptibility to chemically induced mitochondrial damage in cells.^[24]^ Conversely, the activation of Nrf2 by chemoprotectants provides defense against mitochondrial damage. In heat-stress-induced mice, Nrf2 inhibited oxidative damage in the testes.^[25]^ Additionally, Zhang et al^[26]^ demonstrated that melatonin stimulates SIRT1/Nrf2 signaling by activating MT1/MT2, thereby preventing cisplatin-induced apoptosis of Leydig cells. In summary, enhanced NFE2L2 expression was shown to potential protection against cuproptosis in the testes.

The NLRP3 gene, also known as the NOD-like receptor family pyrin domain-containing 3, is involved in multiple cell death pathways, including apoptosis, necroptosis, and ferroptosis. Previous studies have reported elevated NLRP3 levels in patients with varicocele or azoospermia, indicating its potential role in male infertility.^[27]^ It has been found that NLRP3 promotes obesity-related spermatogenesis impairment and is involved in the production of inflammatory factors that can disrupt normal spermatogenic function.^[28]^ Additionally, Poli et al^[29]^ reported that the production of nitric oxide and inflammatory factors can induce the assembly of the NLRP3 inflammasome complex through the differentiation of testis macrophages. This increase in inflammasome activity can subsequently promote the production of reactive oxygen species (ROS) and disrupt the normal spermatogenic function of the testis. Our results also showed the high levels of NLRP3 in NOA patients. Xu et al^[30]^ reported that activation of the Nrf2/ARE pathway activation inhibits NLRP3 inflammasome activation. We observed a positive correlation between NLRP3 and NFE2L2 expression. These findings highlight the potential role of NLRP3 and the Nrf2-NLRP3 axis in the development of NOA through cuproptosis.

The PDHA1 gene encodes for the E1 alpha subunit of the pyruvate dehydrogenase complex, which is responsible for the conversion of pyruvate to acetyl-CoA in the mitochondria. This enzyme plays a crucial role in aerobic metabolism by linking glycolysis to the TCA cycle. The PDHA1 is found in the somatic cells and testes, is mostly expressed in the Sertoli cells, followed by diploid and haploid cells, and is undetectable in the spermatozoa.^[31]^ Tsvetkov et al^[11]^ found that PDHA1 might play a pivotal role in malignancies by regulating cuproptosis. Deng et al^[32]^ found that PDHA1 expression was closely linked to several cancer-associated signaling pathways, such as DNA damage. In our study, GVSA analyses were also showed that higher expression of PDHA1 was associated with DNA repair related pathways.

SLC31A1, also known as copper transporter 1 (CTR1), is responsible for maintaining copper homeostasis by facilitating the cellular uptake of copper from the extracellular environment into cells. CTR1 acts as an ion-gated channel and plays a crucial role in copper transport.^[33]^ CTR1 has been implicated in cisplatin-induced testicular germ cell apoptosis.^[34]^ Ghaffari et al discovered that the uptake of cis-diamminedichloroplatinum (II) (cDDP) by CTR1 in Sertoli cells resulted in the accumulation of cDDP in the testis. This accumulation plays a crucial role in germ cell loss through apoptosis. We observed high expression of SLC31A1 in patients with NOA. This increased expression suggests that there may be an elevated uptake of copper into cells. Consequently, cell death was induced by cuproptosis may occur.

The identification of upstream noncoding RNAs, including miRNAs and lncRNAs, associated with hub CRGs is an important step in understanding the regulatory mechanisms involved in the development of NOA. In this study, 55 miRNAs and 177 lncRNAs were predicted to be associated with these genes. Advancements in biological sequencing technology and the exponential growth of sequencing data have provided valuable resources for bioinformatics analysis. These analyses allow researchers to predict and quantify the associations between non RNAs and diseases, such as NOA.^[35]^ By leveraging big data analysis techniques, researchers can identify the most relevant noncoding RNAs associated with diseases, which can then be validated through subsequent experimental studies.^[36]^

Bioinformatics analysis conducted in this study revealed that the expression of genes related to homologous recombination was downregulated in the NOA group compared to that in control group. Homologous recombination is an essential DNA repair mechanism that maintains genomic stability and ensures the accurate repair of DNA double-strand breaks.^[37]^ Deficiencies in the DNA repair machinery in germ cells can lead to increased DNA damage, which can have detrimental effects on spermatogenesis and fertility.^[38]^ Copper accumulation can induce DNA fragmentation, DNA damage, and base substitution mutants.^[39]^ We found that genes related to homologous recombination were expressed at lower levels in the NOA groups than in the control groups. This deficiency may contribute to DNA damage accumulation in germ cells, leading to abnormal spermatogenesis and infertility. Further investigation of the specific mechanisms underlying this deficiency in homologous recombination could provide valuable insights into the molecular basis of NOA.

Nonetheless, it is important to acknowledge the limitations of this study. While the 6 hub CRGs identified were systematically analyzed using bioinformatics and QPCR in NOA patients, it is crucial to note that the study lacked validation with a large sample size and in-depth investigation of the underlying mechanisms. To address these limitations, future research should expand the study sample size and provide deeper basic research.

## 5. Conclusions

We found that DBT, GCSH, NFE2L2, NLRP3, PDHA1, and SLC31A1 may serve as predictors of cuproptosis and play important roles in NOA. These findings offer valuable insights for future investigations into the underlying mechanisms underlying cuproptosis in NOA.

## Acknowledgments

The authors gratefully acknowledge the data provided by the patients and researchers participating in GEO.

## Author contributions

**Writing – original draft:** Silin Jiang.

**Methodology:** Yong Wei.

**Data curation:** Yongshan Li.

**Validation:** Wei Liu, Zhenzhong Wang.

**Writing – review & editing:** Xuhui Meng, Luming Shen.

**Supervision:** Qingyi Zhu.

## Supplementary Material


