# Supplementary material for: A comparative cross-platform analysis of cuproptosis-related genes in human nonobstructive azoospermia: An observational study
Source: Medicine (Baltimore). 2024 Aug 2;103(31):e39176. doi: 10.1097/MD.0000000000039176 (PMC11296415; doi:10.1097/MD.0000000000039176)
Supplement: Supplementary file 1 [file medi-103-e39176-s001.docx]

Table S1. Sequences of primers for qRT-PCR.

| Gene |  | Primer Sequence (5'-3') |
| --- | --- | --- |
| DBT | Forward | CTCCGGTATTCACAGGCAAAG |
|  | Reverse | AAAATGAGGTATCTTCAGGGCTG |
| GCSH | Forward | GGAAGCGTTGGGAGATGTTGT |
|  | Reverse | TCTGAAGGGTTACTCAGTGTCA |
| NFE2L2 | Forward | TCAGCGACGGAAAGAGTATGA |
|  | Reverse | CCACTGGTTTCTGACTGGATGT |
| NLRP3 | Forward | GATCTTCGCTGCGATCAACAG |
|  | Reverse | CGTGCATTATCTGAACCCCAC |
| PDHA1 | Forward | TGGTAGCATCCCGTAATTTTGC |
|  | Reverse | ATTCGGCGTACAGTCTGCATC |
| SLC31A1 | Forward | GGGGATGAGCTATATGGACTCC |
|  | Reverse | TCACCAAACCGGAAAACAGTAG |
| GAPDH | Forward | GGAGCGAGATCCCTCCAAAAT |
|  | Reverse | GGCTGTTGTCATACTTCTCATGG |
